# Lactate transport and receptor actions in cerebral malaria

**DOI:** 10.3389/fnins.2014.00125

**Published:** 2014-05-19

**Authors:** Shelton T. Mariga, Miriam Kolko, Albert Gjedde, Linda H. Bergersen

**Affiliations:** ^1^Department of Neuroscience and Pharmacology, University of CopenhagenCopenhagen, Denmark; ^2^Department of Ophthalmology, Roskilde HospitalRoskilde, Denmark; ^3^The Brain and Muscle Energy Group and SN-Lab, Department of Anatomy and Department of Oral Biology, Institute of Basic Medical Sciences and Centre for Molecular Biology and Neuroscience/SERTA Healthy Brain Aging Centre, University of OsloOslo, Norway

**Keywords:** energy metabolism, cerebral malaria, lactate transport, lactate receptor, volume transmitter

## Abstract

Cerebral malaria (CM), caused by *Plasmodium falciparum* infection, is a prevalent neurological disorder in the tropics. Most of the patients are children, typically with intractable seizures and high mortality. Current treatment is unsatisfactory. Understanding the pathogenesis of CM is required in order to identify therapeutic targets. Here, we argue that cerebral energy metabolic defects are probable etiological factors in CM pathogenesis, because malaria parasites consume large amounts of glucose metabolized mostly to lactate. Monocarboxylate transporters (MCTs) mediate facilitated transfer, which serves to equalize lactate concentrations across cell membranes in the direction of the concentration gradient. The equalizing action of MCTs is the basis for lactate’s role as a volume transmitter of metabolic signals in the brain. Lactate binds to the lactate receptor GPR81, recently discovered on brain cells and cerebral blood vessels, causing inhibition of adenylyl cyclase. High levels of lactate delivered by the parasite at the vascular endothelium may damage the blood–brain barrier, disrupt lactate homeostasis in the brain, and imply MCTs and the lactate receptor as novel therapeutic targets in CM.

## CEREBRAL MALARIA

Malaria is caused by the protozoan parasite *Plasmodium*. About two million children die each year world-wide (Breman, 2001), mainly due to severe complications, especially from cerebral malaria (CM), of *Plasmodium falciparum* infection, the majority in sub-Saharan Africa, but also in Southeast Asia and South America. In developed countries, CM affects mainly returning travelers. Brain edema, lactate accumulation, and intracranial hypertension are important characteristics of CM, and untreated CM often leads to coma and death within 24 h (Newton and Krishna, 1998). CM survivors sometimes suffer from general disorders such as acidosis, as well as more specific neurological disorders such as ataxia, epilepsy, and blindness. However, the pathogenesis of CM remains unclear. Despite intense research, no effective vaccine is yet available, and the problem of drug-resistant malaria is increasing (Mariga et al., 2004).

## CM FROM CIRCULATING ASEXUAL *Plasmodium falciparum*

The *Plasmodium* parasite invades erythrocytes as part of its asexual life cycle within the human host. It matures within these blood cells (*Plasmodium*-invaded red blood cells, PRBC) to the trophozoite and schizont stages. It then disappears from the peripheral circulation and is sequestrated in the vascular beds of critical organs such as the brain (Kristensson et al., 2013). Progression to CM is closely linked to erythrocytes containing mature stages of *P. falciparum* confined in the brain microvasculature (Dorovini-Zis et al., 2011) and host immunocompentent cells targeting the malaria parasite (Idro et al., 2005). Mechanisms by wish an intraerythrocytic parasite lodged in the vascular space of the brain can elicit such dramatic neurological effects necessarily involve local and systemic metabolic intermediates, as well as the transport systems for water and metabolites that link the membranes of the PRBC to the blood–brain barrier (BBB) and the end-feet of astrocytes.

## DYNAMICS OF BLOOD AND BRAIN LACTATE IN CM

Several factors contribute to the accumulation of lactate in brain tissue in CM. The lack of a functional citric acid (TCA) cycle in human and murine *Plasmodium* parasites makes them largely reliant on glycolysis to fulfill their very substantial energy requirements (Sherman, 1998). Erythrocytes infected with mature parasites at the trophozoite stage consume glucose two orders of magnitude faster than normal, uninfected erythrocytes (Scheibel et al., 1979; Roth et al., 1982) with commensurate generation of lactic acid (Pfaller et al., 1982) i.e., at 5–100 times the rates of uninfected erythrocytes (Zolg et al., 1984). The resulting quantities of lactate leave the cells as directed by the concentration gradient. At rest, lactate in brain also is transported from brain tissue to plasma, but given the high rate of release of lactate from *Plasmodium* infected erythrocytes sequestered on the endothelial cells, lactate generated by parasites moves from the plasma to brain tissue down a concentration gradient.

## CEREBRAL LACTATE CONTENT AND TRANSPORT

The quantities of lactate produced by the parasites compete with the lactate generated in the host’s own brain metabolism for transport to or from the brain tissue. A system of transporters of lactate is present at the parasite surface as H^+^^+^-coupled monocarboxylate transporters (MCTs) with features in common with members of the MCT family of higher eukaryotes (Kanaani and Ginsburg, 1991, 1992; Elliot et al., 2001). The MCT family members are well characterized in human brain (Bergersen et al., 1999, 2001; Pierre and Pellerin, 2005; Bergersen, 2007; Lauritzen et al., 2013a,b). In general, MCT1 is at the vascular endothelium, MCT4 in astrocytes, and MCT2 on neurons. Microglia expresses MCT1 and MCT2 after cerebral ischemia (Moreira et al., 2009). Both the MCTs and the lactate receptor GPR81 display affinities for L-lactate in the low mM range (Cai et al., 2008) that are consistent with the range of extracellular lactate concentrations measured by microdialysis in the brain tissue *in vivo* in human subjects (Abi-Saab et al., 2002; Jalloh et al., 2013).

## LACTATE FLUX RECEPTOR ACTION AT THE BLOOD–BRAIN BARRIER

The BBB is constituted by the tight junctions of the endothelium of cerebral microvessels and capillaries. Interaction between lactate and lactate transport systems is likely to be perturbed at the BBB that is an important site of CM pathogenesis according to data from human autopsies, animal models, and *in vitro* cell cultures (Tripathi et al., 2007). At the BBB interfaces between the brain parenchyma and the parasite infected erythrocytes, any impairment of the integrity of endothelial cells of the cerebral microvasculature will lead to cerebral edema and lactic acidosis by the disruption of ion and solute homeostasis that follows increased membrane permeability. Normally, lactate enters the brain by first being transported through the endothelial cells and then diffusing extracellularly in the brain interstitial space and intracellularly after transport by the MCTs. Lactate down-regulates cAMP by interacting with GRP81, which is exposed on the luminal as well as the abluminal membranes of the endothelial cells and to a lesser extent on the membranes of astrocytes (Lauritzen et al., 2013a,b).

## CM ASSOCIATION WITH ELEVATED LACTATE

Association of CM with increased CSF (cerebral spine fluid) lactate is now well established. As a signaling molecule as well as a substrate for energy metabolism, brain lactate is an important diagnostic marker in clinical settings. Lactate concentrations can be used to monitor disease progress and during trials of drug effects in CM. Results from clinical settings and animal models provide convincing evidence of increased lactate concentrations both in extracellular fluid and CSF in CM. Several investigators confirmed lactate as the single best indicator of prognosis (White et al., 1985; Krishna et al., 1994) when measured in CSF of patients infected with *P. falciparum* (Tosti et al., 2007). In the patients, CSF concentrations correlated with the state of the disease (White et al., 1985), with death observed at levels higher than 6–9 mM lactate. Survivors always had less than 3.4 mM.

More than 80% of children from Malawi, Kenya, and Gambia with CM (Waller et al., 1991) with elevated CSF pressure revealed 40% higher lactate concentrations compared to normal (Newton et al., 1994; Potchen et al., 2012). The combination of elevated pressure and elevated lactate often is fatal (Newton et al., 1994, 1997), with most autopsies revealing high brain lactate concentrations (SenGupta and Naraqi, 1992; Walker et al., 1992). In adult Thai patients, CNS lactate in CSF at twice the normal value was associated with 100% mortality, suggestive of metabolic disturbances in the brain, indicative of a fundamental pathological process (Dorovini-Zis et al., 2011). Declining lactate during CM is associated with recovery, confirming that severe lactacidosis is a reliable indicator of brain damage, (Newton, 2005). Similar lactate profiles are observed in murine CM models, when brain lactate increases a few days post-infection (Sanni et al., 2001).

## ASSOCIATION WITH MALARIA RETINOPATHY AND LACTATE

Malaria retinopathy occurs in approximately two-thirds of pediatric patients with CM (Beare et al., 2004, 2006). It is characterized by retinal whitening, vessel changes and retinal hemorrhages. Correlation between malaria retinopathy and the severity of clinical CM symptoms has consistently been shown, and examination of the retina by ophthalmoscopy is useful for diagnosing CM (Beare et al., 2011). Increased plasma lactate levels are associated with the severity of retinopathy and CM, suggesting that the microvascular obstruction observed in the retina reflects systemic and cerebral microvascular obstruction and focal ischemia (Maude et al., 2009a). The eye is an easily examined, and the retina is developmentally a part of the brain and its vasculature a continuation of the cerebral vasculature. Consequently there is increasing attention on retinal pathology in malaria, with the perspective of diagnosis as well as of understanding mechanisms in CM (Maude et al., 2009b; White et al., 2009; Beare et al., 2011). Yet, the cascade of reactions involving lactate and its relation to malaria retinopathy is not fully understood.

## MOUSE MODELS OF CM

The present perspective focuses on the critical role of lactate in the etiology and development of CM, although CM likely has a multifactorial pathogenesis. Murine models are critical to the study of mechanisms of underlying symptoms observed in CM (Hunt and Grau, 2003; Rénia et al., 2010; Riley et al., 2010). In humans, the signs typically encountered with CM include lactacidosis, seizures, brain edema (Polder et al., 1992; Potchen et al., 2012), and coma, followed by death (Rest, 1982; Hunt and Grau, 2003; Medana and Turner, 2006). Lactate accumulation is associated with decreased brain perfusion and brain hypoxia in a mouse model (Promeneur et al., 2013). These observations indicate intravascular obstruction and reduced perfusion of brain microvessels in CM.

To resolve the mechanisms of these changes, it is necessary to determine the cellular expression, localization and potential redistribution of the brain MCTs (MCT1, MCT2, MCT4), as well as the lactate receptor (GPR81), to reveal the dynamics of lactate’s role in CM. This is possible with the mouse models of CM, established by infection with the rodent malaria parasite *P. berghei* ANKA in susceptible strains, C57BL/6 (Rest, 1982) or CBA/J (Penet et al., 2005). *In vivo* studies of the latter model demonstrated massive brain edema coupled to lactacidosis associated with compression of cerebral arteries, ultimately causing coma and death, but no attempt was made to study the brain lactate transport systems (Penet et al., 2005). Using the CM C57BL/6 mouse model, Promeneur and collaborators (Promeneur et al., 2013) found that the water channel aquaporine 4 (AQP4) was downregulated in CM. Also, knock-out C57BL/6 mice lacking AQP4 succumbed earlier to *P. berghei* ANKA infection and were more severely affected than wild type mice, indicating that the water channel is protective in CM. It is particularly important to repeat these experiments in GPR81 knock-out mice to determine whether volume transmission followed by remote action at the lactate receptor is an important factor in CM.

## MCT AND GPR81 IN CM

In the brain and retina, MCTs, like AQP4, are highly expressed in ependymal cells and astrocytes with polarized distribution in end-feet membranes facing cerebral capillaries and pia mater (Bergersen, 2007). GPR81 has a similar distribution (Lauritzen et al., 2013a,b). MCTs have also been identified in retinal sections (Bergersen et al., 1999) and unpublished results reveal GPR81 staining in human retinal sections (Kolko and Bergersen, in preparation). In addition MCT1 and GPR81 are highly expressed in the endothelial cells. The strategic locations at the blood–brain and cerebrospinal fluid–brain interfaces indicate that MCTs and GPR81 are major factors in the regulation of lactate movement into and out of the brain. Such movements were suggested already by early *in vivo* work (Gjedde et al., 1975) and are now firmly established (van Hall et al., 2009). Lactate receptor actions have been demonstrated in brain, reducing cAMP (apparent IC_50_ 29 mM; Lauritzen et al., 2013a,b) and neuronal calcium spiking (apparent IC_50_ 4.2 mM; Bozzo et al., 2013) through GPR81 activation. In addition, lactate stimulates locus coeruleus neurons (apparent IC_50_ 0.7 mM; Tang et al., 2014), ascribed to a receptor other than GPR81. These observations and the volume transmitter role of lactate (Bergersen and Gjedde, 2012), whereby lactate diffuses through large volumes of brain tissue to produce distant receptor effects, therefore imply actions of lactate produced by malaria parasites as important factors in CM.

Epileptic seizures occur in more than 70% of CM patients on admission (Newton et al., 2000; Idro et al., 2010). Elevated lactate concentrations in the brain are highly correlated with the severity of repeated seizures commonly observed in CM. Similarly, seizures in temporal lobe epilepsy (TLE) with hippocampal sclerosis, known as mesial temporal lobe epilepsy (MTLE), are correlated with high lactate levels as observed by microdialysis in epileptic patients (During et al., 1994). Brain MCTs expressions and distributions might be influenced by multiple forms of brain pathology including MTLE, stroke, meningitis, neuromyelitis optica, and brain tumors (Bergersen, 2007; Lauritzen et al., 2011, 2012, 2013a,b). Observations in MTLE and other neuropathological disorders resemble in some respects, the CM-related clinical and pathological status (i.e., seizures, lactacidosis). However, the role of lactate and the receptor action and transport in CM and other disease states need to be clarified.

## HYPOTHESIS OF A NEW FACTOR IN CM

**FIGURE 1:**
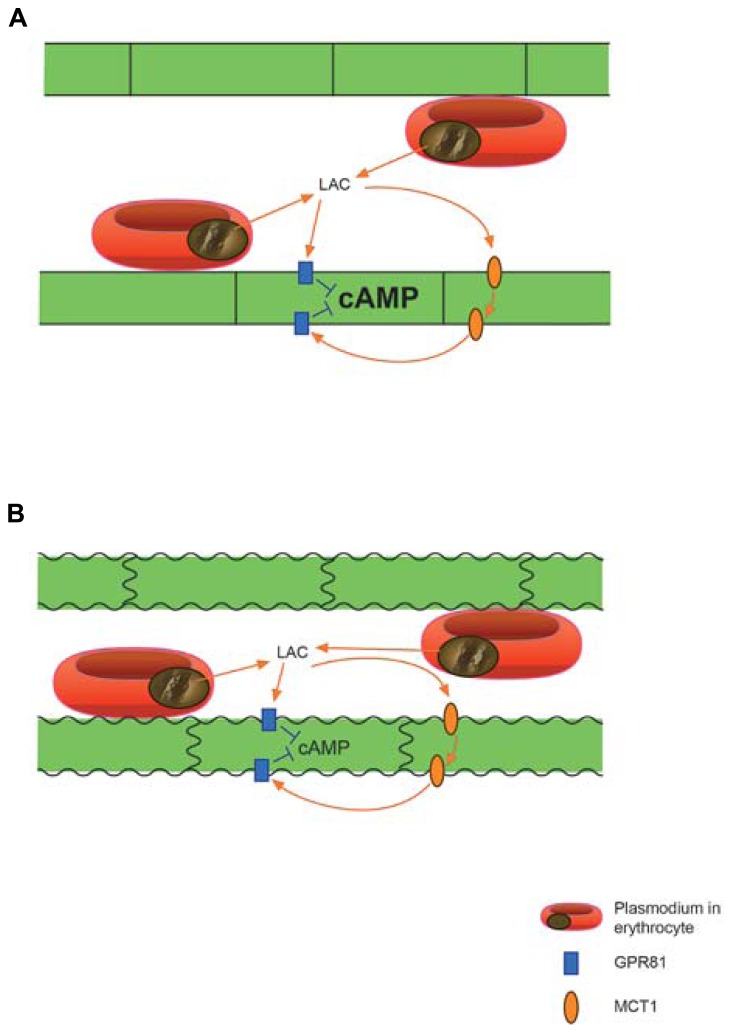
**Proposed action of extreme quantities of *Plasmodium* derived lactic acid on brain vascular endothelium. (A)** Lactate penetrating the endothelial cells through MCT1 activates the lactate receptor GPR81, which is situated on both sides of the endothelium, to cause down-regulation of cAMP by inhibiting adenylyl cyclase activity. **(B)** Reduced levels of cAMP can lead to vasoconstriction and endothelial dysfunction with weakening of cell–cell junctions ultimately causing break-down of the blood-brain-barrier. Not shown: the GPR81 mediated effects may act in concert with pH effects of lactic acid and with other deleterious agents from the protozoon. Lactate penetrating the endothelium may also act on GPR81 expressed on neurons and to a lesser degree on astrocytic perivascular end-feet.

Based on the extant evidence, we claim that the lactate transporters and the lactate receptor GPR81 together are an essential factor in the pathophysiology of CM. The most obvious scenario (**Figure 1**) under this hypothesis is that lactate, profusely released from *Plasmodium* infected erythrocytes sequestered at the capillary endothelium, excessively down-regulates cAMP via GPR81. High concentrations of lactate will reach receptors both at the luminal and abluminal surface of the endothelial cells, because of the presence of MCT1 (Lauritzen et al., 2011) as well as GPR81 (Lauritzen et al., 2013a,b) at both sides of the endothelium. Normally as well as in disease, cAMP is perhaps the most potent signaling molecule to stabilize the endothelial barrier, and also regulates inflammation response and vascular tone (Roberts and Dart, 2014; Schlegel and Waschke, 2014). It acts on three classes of proteins: protein kinase A (PKA), cyclic-nucleotide-gated ion channels, and Epacs (exchange proteins directly activated by cAMP). The Epacs constitute a family of guanine-nucleotide-exchange factors for the small Ras-related GTPases Rap1 and Rap2. Both PKA and Rap1 can cause Rac1-mediated strengthening of adherens junctions and of the actin cytoskeleton (Schlegel and Waschke, 2014).

Extreme down-regulation of cAMP can therefore be expected to cause deterioration of the endothelium, including break-down of regulated trans-endothelial transport and of the junctions between endothelial cells, i.e., break-down of the BBB (**Figure 1**). In addition, increased vascular tone (Roberts and Dart, 2014) may aggravate the hypoperfusion caused by clogging of microvessels by sequestered erythrocytes. The effects via GPR81, and possibly other lactate receptors (cf. Tang et al., 2014), may converge with effects of acidification caused by proton from the parasite produced lactic acid, as well as with other deleterious compounds originating in the *Plasmodium* infected erythrocytes (Aird et al., 2014).

Within the brain parenchyma, excessive lactate concentrations delivered by the parasites must be expected to perturb the volume transmitter function of lactate (Bergersen and Gjedde, 2012), although the concentration of lactate will decrease with the distance from the parasites, which are confined to the vasculature. The vascular end-feet processes of astrocytes have relatively low GPR81 concentrations, while the synaptic membranes of excitatory synapses have the highest concentrations of GPR81 (Lauritzen et al., 2013a,b), but are at a greater distance from the parasites. The effects of excessive lactate in the parenchyma are harder to predict than those on the BBB, but they are expected to be exacerbated in CM, because lactate induced down-regulation of cAMP will upregulate MCT1 at the endothelial plasma membrane (Smith et al., 2012).

Inflammatory responses through the innate immune system may serve to confine the infection or cause serious tissue damage, the relative weight of these effects depending on genetic characteristics of the *Plasmodium* and of the host (Wu et al., 2014). Careful dissection of the mechanisms may reveal ways to augment antiparasite mechanisms but suppress tissue damage. A recent paper (Hoque et al., 2014) found that lactate by activating GPR81 reduced tissue injury in disease models. The fact that this effect was mediated down stream through arrestin beta-2 rather than cAMP predicts the possibility of separately modifying deleterious and beneficial effects of GPR81 activation.

Our hypothesis may be tested in mouse models of CM. The role of GPR81 can be ascertained by inducing CM in GPR81 knock-out mice. This will reveal whether the lack of the lactate receptor confers resistance to CM and/or ameliorates the pathology and outcome, in which case GPR81 antagonists would offer novel treatment for CM. GPR81 antagonists are not available at present, but may soon be. Further, MCT1 blockers, which are presently introduced in cancer therapy (Parks et al., 2013; Draoui et al., 2014) may slow the release of lactate from the infected erythrocytes and its transfer across the endothelium. If so, they would offer another novel therapy against CM. The existence of MCT blockers that selectively inhibit lactate influx but not efflux (Draoui et al., 2014) would allow the action to be restricted to the luminal side of the endothelium, avoiding possibly deleterious actions of the drugs on the brain parenchyma, which may also be avoided by using GPR81 and MCT antagonists that are hydrophilic and do not penetrate the BBB. The hypothesis can be further tested by analysis of MCT and GPR81 mRNAs and protein expressions and by immunohistochemistry by light and electron microscopy of the brain in CM mice and non-CM controls. It is particularly important to examine the hippocampal formation, in view of its central role in epileptogenesis and in view of the demonstrated roles of MCTs in human epilepsy and animal models of TLE (Lauritzen et al., 2013a,b). In addition, retinal sections must be examined to identify the expression pattern of MCTs and GPR81 in CM mice. The possible impact of antimalarial drugs on lactate transport and receptor activity should be tested.

## CONCLUSION

Studies on lactate transport and receptor action in CM are expected to provide important new insights into the pathogenesis of CM, and to identify novel therapeutic targets.

## Conflict of Interest Statement

The authors declare that the research was conducted in the absence of any commercial or financial relationships that could be construed as a potential conflict of interest.
